# Prevasculitic Eosinophilic Granulomatosis With Polyangiitis

**DOI:** 10.7759/cureus.14649

**Published:** 2021-04-23

**Authors:** Kathryn M Burtson, Jonathan Bishop

**Affiliations:** 1 Internal Medicine, Wright Patterson Air Force Base/Wright State University, Dayton, USA; 2 Critical Care Medicine, University of Pittsburgh Medical Center Mercy, Pittsburgh, USA

**Keywords:** churg-strauss syndrome, vasculitis, prevasculitic stage, eosinophilia, eosinophilic gastritis, eosinophilic duodenitis

## Abstract

Eosinophilic granulomatosis with polyangiitis (EGPA) is an exceptionally rare systemic necrotizing vasculitis. The disease is clinically characterized by asthma with concomitant blood and tissue eosinophilia, often progressing to eosinophilic vasculitis. From the onset of asthma, there is usually a three to nine year delay of EGPA diagnosis. We report a case of this highly uncommon disease identified in an early stage.

## Introduction

Eosinophilic granulomatosis with polyangiitis (EGPA) is a systemic necrotizing vasculitis clinically characterized by asthma with concomitant blood and tissue eosinophilia, often progressing to small vessel eosinophilic vasculitis [1,2]. EGPA, formally known as the Churg-Strauss syndrome, was first described in Mount Sinai Hospital, New York in 1951 by Jacob Churg and Lotte Strauss. It was recognized after studying a series of 13 patients who had severe asthma, fever, and hypereosinophilia, together with symptoms of vascular involvement in various organ systems [1].

EGPA is exceptionally uncommon, with an annual incidence of 1-3 per million. EGPA has an overall prevalence of 14 per 1,000,000 adults. The mean age of onset is 48 years. A three to nine year delay from the development of asthma to EGPA diagnosis has been reported [2]. EGPA has an initial, prevasculitic, stage characterized with eosinophilic tissue infiltration and an absence of overt vasculitis. We report a case of this extremely uncommon disease identified in an early stage.

## Case presentation

The patient was a healthy 41-year-old male who initially presented to the outpatient clinic with typical rhinosinusitis, unresponsive to conventional therapy. Nasal polyposis was identified requiring surgical excision, and the tissue pathology was eosinophil predominant.

**Figure 1:**
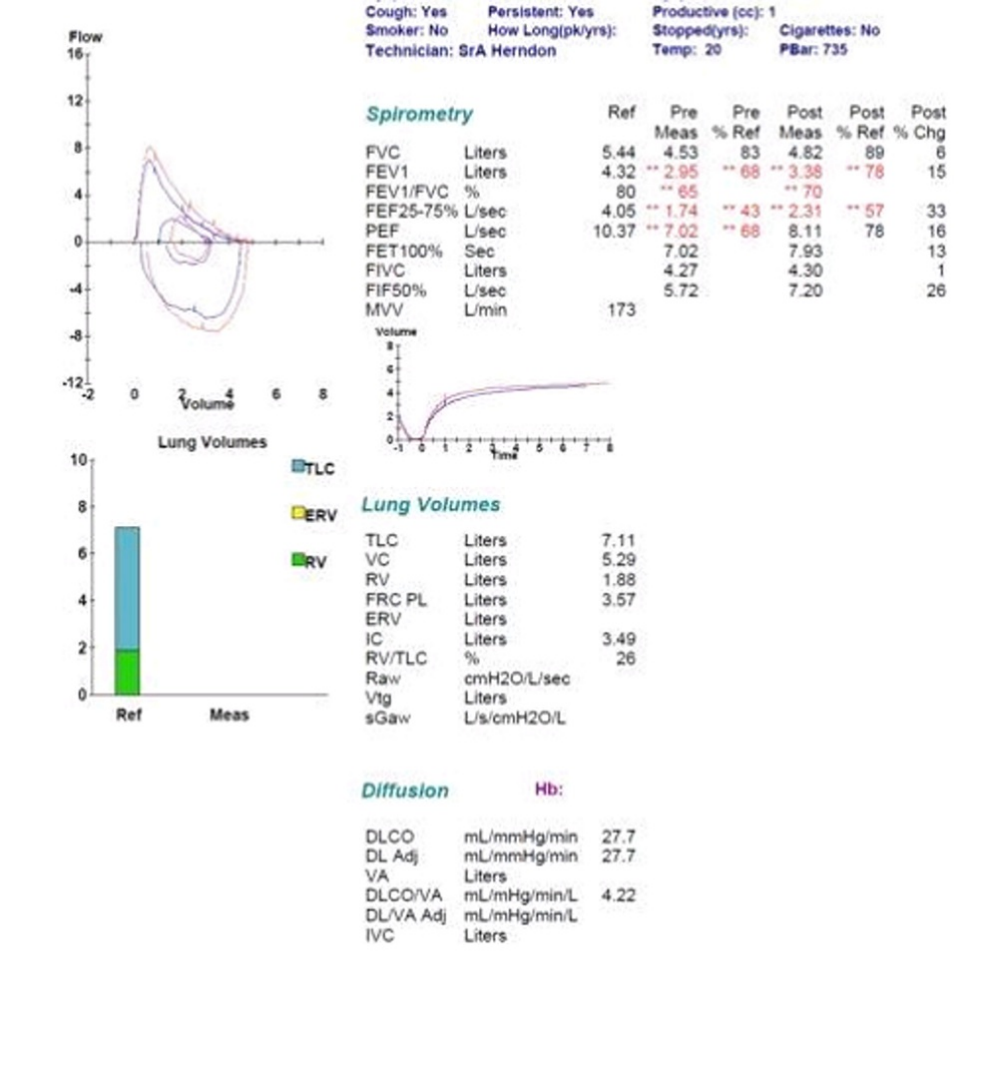
Plethysmography report.

Later, he developed acute eosinophilic cholecystitis prompting cholecystectomy. Post-operatively, the patient developed acute bronchospasm requiring reintubation and initiation of systemic corticosteroids with intravenous methylprednisolone. Throughout the hospital course patient was found to have eosinophilia on daily complete blood count evaluation. The patient’s clinical status improved and he was extubated. Later, he was discharged home on a steroid taper. As an outpatient, he underwent further diagnostic evaluation with bone marrow biopsy, which was negative for leukemia and hypereosinophilic syndrome. Pulmonary function tests revealed moderate obstructive lung disease (forced expired volume in one second 68% predicted; 15% bronchodilator response) (Figure 1).

**Figure 2:**
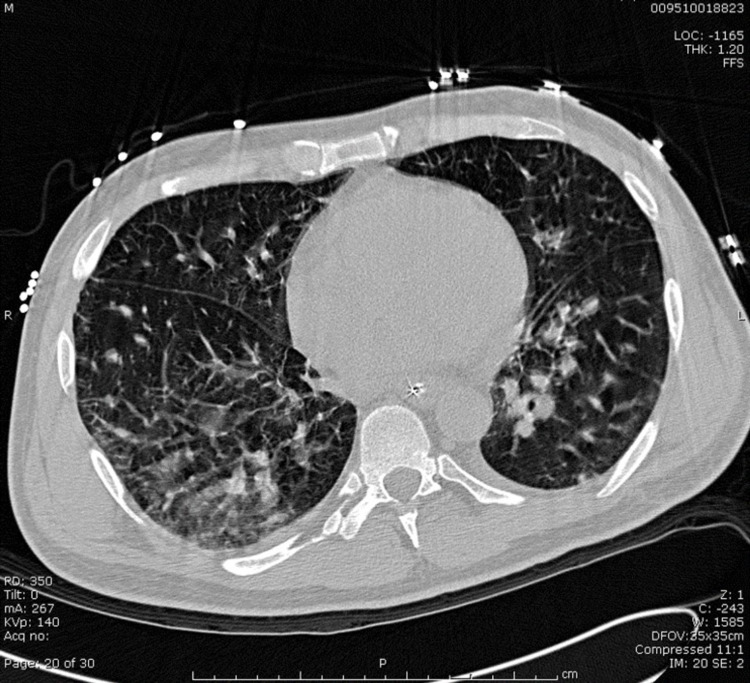
Patchy pulmonary noninfectious infiltrates.

**Figure 3:**
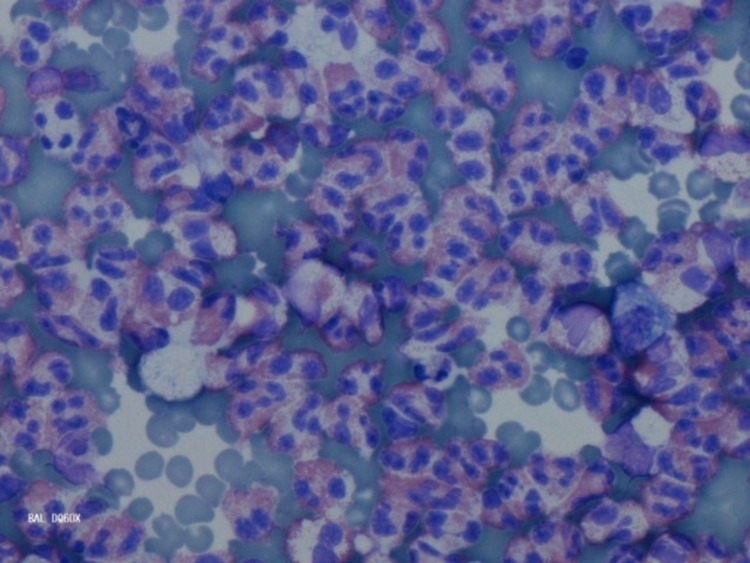
Bronchoalveolar lavage with 93% eosinophils.

**Figure 4:**
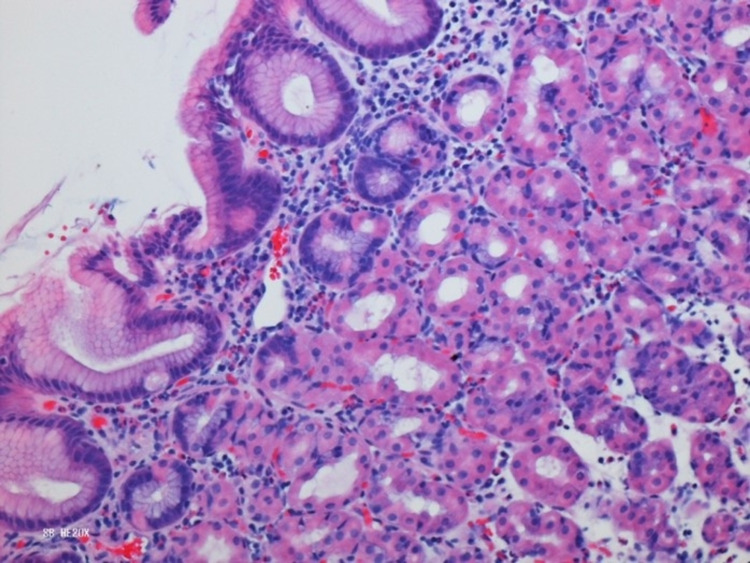
Eosinophilic gastritis.

**Figure 5:**
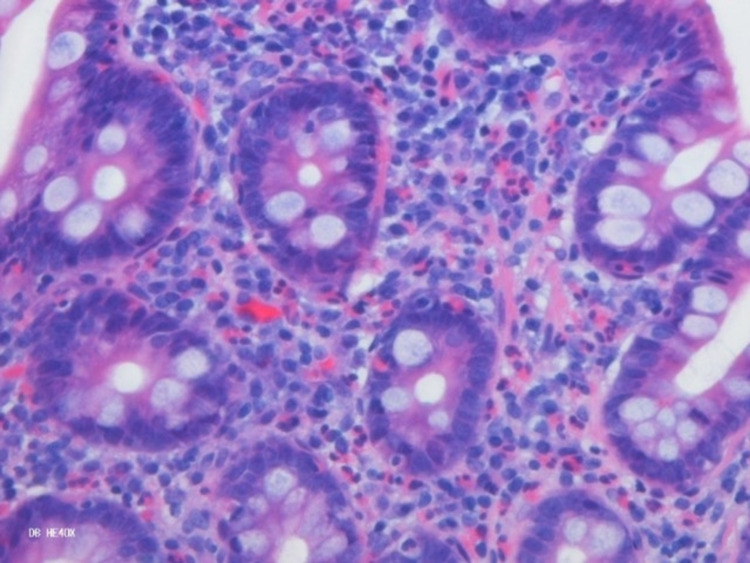
Eosinophilic duodenitis.

Upon completing the steroid taper, the patient presented to the hospital with abdominal pain, nausea, and vomiting prompting an admission to the medical service. His peripheral eosinophilia (total eosinophils: 1,400/mcL) had become more prominent. Laboratory studies were notable for elevation of serum immunoglobulin E to 1,014 IU/mL and weakly positive rheumatoid factor (1:32); antineutrophil cytoplasmic antibodies and antinuclear antibody panels were negative. Computed tomography of the lungs demonstrated new patchy pulmonary noninfectious infiltrates (Figure 2), which a subsequent bronchoscopy demonstrated to be noninfectious in origin, with bronchoalveolar lavage notable for the presence of 93% eosinophils (Figure 3). Biopsy results from colonoscopy and esophagogastroduodenoscopy were consistent with eosinophilic gastritis (Figure 4) and duodenitis (Figure 5) without evidence of vasculitis. The patient was initiated on high-dose corticosteroids with a continued taper, resulting in complete remission of all of his signs and symptoms of EGPA.

## Discussion

The patient satisfied four out of six American College of Rheumatology criteria for diagnosis of EGPA, including asthma, peripheral eosinophilia, mono or polyneuropathy (not present), pulmonary infiltrates, paranasal sinusitis, and eosinophilic vasculitis (not present). A diagnosis of EGPA can be made if at least four of the six criteria are met. These diagnostic criteria have a sensitivity of 85% and a specificity of 99.7% [3].

The early symptoms of EGPA include nonspecific constitutional symptoms such as myalgias, malaise, fever, and weight loss. There are three distinct but not always sequential phases of this disease. The first is a prodromal phase dominated by upper and lower respiratory manifestations such as asthma, rhinosinusitis, and nasal polyposis. The adult-onset asthma of EGPA is typically refractory to traditional treatments and grows increasingly severe over time. The second phase, characterized by blood and tissue eosinophilia, represents the prevasculitic phase. Histologically, the first two phases are characterized by extensive eosinophilic infiltration. The third is the vasculitic phase. Granulomas and necrotizing vasculitis histologically characterize the third phase. Peripheral blood eosinophilia (>10% differential on white blood cell count) is one of the hallmarks of EGPA and may characterize any stage of the disease [4].

Prevasculitic EGPA is usually treated with high-dose corticosteroids, with this stage of the disease typically exceptionally responsive to this therapy [4]. Initial treatments consist of prednisone at a dosage of 1 mg/kg followed by a prolonged taper [2]. Later stages of the disease may require immunosuppressant adjuncts for severe manifestations. Novel targeted therapies are being studied for patients with relapses, refractory disease, or sequela of corticosteroid dependence [5].

EGPA is an exceptionally rare systemic necrotizing vasculitis. The disease is clinically characterized by asthma with concomitant blood and tissue eosinophilia, often progressing to eosinophilic vasculitis [1,2]. From the onset of asthma, there is usually a three to nine year delay of EGPA diagnosis. EPGA mortality is the lowest of all forms of the anti-neutrophilic cytoplasmic autoantibody-associated vasculitides, with a five-year survival of 91% [6].

## Conclusions

EGPA is an exceptionally rare systemic necrotizing vasculitis. The disease is clinically characterized by asthma with concomitant blood and tissue eosinophilia, often progressing to eosinophilic vasculitis. We reported a case of this extremely uncommon disease identified in an early stage.

## References

[1] Churg J, Strauss L (1951). Allergic granulomatosis, allergic angiitis, and periarteritis nodosa. Am J Pathol.

[2] Baldini C, Talarico R, Della Rossa A, Bombardieri S (2010). Clinical manifestations and treatment of Churg-Strauss syndrome. Rheum Dis Clin North Am.

[3] Masi AT, Hunder GG, Lie JT (1990). The American College of Rheumatology 1990 criteria for the classification of Churg-Strauss syndrome (allergic granulomatosis and angiitis). Arthritis Rheum.

[4] Churg A (2001). Recent advances in the diagnosis of Churg-Strauss syndrome. Mod Pathol.

[5] Raffray L, Guillevin L (2018). Treatment of eosinophilic granulomatosis with polyangiitis: a review. Drugs.

[6] Tsurikisawa N, Oshikata C, Kinoshita A, Tsuburai T, Saito H (2017). Longterm prognosis of 121 patients with eosinophilic granulomatosis with polyangiitis in Japan. J Rheumatol.

